# Lung Point of Care Ultrasound (POCUS) in Cardiorespiratory Physiotherapy and Respiratory Therapy Practices: Current Status and Future Directions

**DOI:** 10.24908/pocus.v9i2.17854

**Published:** 2024-11-15

**Authors:** George Ntoumenopoulos, Georgina A Pizimolas, Sarine Mani, Simon Hayward, Jane Lockstone

**Affiliations:** 1 Department of Physiotherapy, Royal Perth Hospital Perth AUS; 2 Department of Physiotherapy, The Queensland Children's Hospital Brisbane AUS; 3 Department of Physiotherapy, Launceston General Hospital Tasmania AUS; 4 Department of Physiotherapy, Blackpool Victoria Hospital Blackpool GBR

**Keywords:** Physical Therapists, Respiratory Therapists, Ultrasound, Intensive Care Units, Clinical Decision-Making, Physical Therapy Modalities

## Abstract

Lung Point of Care Ultrasound (POCUS) strongly influences physiotherapy and respiratory therapy clinical decision-making in the intensive care unit (ICU). The uptake of Lung POCUS training by physiotherapists and respiratory therapists is low in some countries, often due to many barriers to its implementation. The safe and appropriate integration of Lung POCUS into physiotherapy and respiratory therapy clinical practice may be achieved by various means. This article describes the pathway for physiotherapists to become Lung POCUS-accredited in Australia, focusing on a novel fast-track approach that may apply internationally and in other professions, such as respiratory therapy. While a fast-track approach is resource intensive, such resource allocation may be justified to support the growth of Lung POCUS in physiotherapy and respiratory therapy practice where mentor support may be inconsistent, lacking, or absent.

## Background

Physiotherapists and respiratory therapists are integral to the multidisciplinary management of patients in the intensive care unit (ICU) 1, 2. Lung Point of Care Ultrasound (POCUS) is an emerging diagnostic tool for respiratory physiotherapists and respiratory therapists, and is suggested to strongly influence clinical decision-making in the ICU 3, 4. Lung POCUS has excellent diagnostic accuracy when compared to other commonly used assessment tools, including lung auscultation and chest x-rays 5, 6. However, like any tool, Lung POCUS is dependent on operator competence. For accurate image acquisition and interpretation, adequate and appropriate training is essential 7. 

In recent years, the United Kingdom (UK) has had a rapid increase in the number of accredited physiotherapists using Lung POCUS (n=205). Now, there is gaining interest in Lung POCUS accreditation and growing Lung POCUS implementation into physiotherapy practice in the UK (personal communication).However, anecdotally, the uptake in Australia appears slower and the number of Lung POCUS-accredited physiotherapists in Australia remains very low (n=10). Lung POCUS training may not be included in the current training programs for physiotherapists and respiratory therapists 8, 9. However, short one-day training programs seem to be effective in improving short-term Lung POCUS knowledge acquisition 10. The implementation of Lung POCUS by respiratory therapists specifically is unclear. A recent survey of 304 therapists (including physiotherapists and 9% respiratory therapists) indicated that the majority of respondents (84%) see Lung POCUS becoming an increasing part of standard assessment in ICU, but only 30% of these therapists reported using Lung POCUS 11. However, of concern, 45% of these Lung POCUS users did not have any formal accreditation in Lung POCUS 11, similar to findings for respiratory therapists from mainland China 9. Across the world, five formal programs exist for critical care ultrasound (predominantly for physicians), including the American College of Chest Physicians, the Society of Critical Care Medicine, the Canadian Intensive Care Society, Focussed Ultrasound in Intensive Care as part of Intensive Care Society United Kingdom (inclusive of non-physician training), and the European Society of Intensive Care Medicine, where lung and vascular ultrasound are the most well-established 12. However, the lack of trainers, time and an agreed-upon set of competencies are the main barriers to the delivery of high-quality training in critical care ultrasound 12. Therefore, the barriers to Lung POCUS competencies such as time and lack of trainers needs to be addressed to improve the implementation of Lung POCUS into physiotherapy and respiratory therapy clinical practice.

### Governance of Lung POCUS in Physiotherapy/Respiratory Therapy Clinical Practice 

A framework for Lung POCUS-use by physiotherapists comprising the elements of (i) scope of practice, (ii) education and competency, and (iii) governance has been published.^13^ This framework ensures the safe and appropriate integration of Lung POCUS into clinical practice by adequately trained and accredited physiotherapists. This framework is also applicable to respiratory therapists, but it may require modifications due to the potential variations in specific professional autonomy, insurance/regulatory arrangements, and accepted practice. In addition, for appropriate governance oversight, hospital and/or department policies and guidelines are needed for quality assurance about imaging practice, archiving/documentation, safety, and professional development. Department managers unfamiliar with POCUS generally, or Lung POCUS specifically, may require reassurance that the appropriate education, training and governance is in place for physiotherapists and respiratory therapists who implement Lung POCUS into their practice.

### Physiotherapy Lung POCUS Accreditation Pathway in Australia 

For a physiotherapist to become formally accredited in Lung POCUS in Australia, there is currently one available pathway. The Australasian Society of Ultrasound in Medicine (ASUM) awards a Certificate in Allied Health Performed Ultrasound (CAHPU), which is available to physiotherapists who are not imaging specialists but who may use Lung POCUS as an imaging tool at the point of care for clinical practice. 

For a physiotherapist to be eligible to enrol in the CAHPU pathway, they must be (i) registered with the Australian Health Practitioner Regulation Agency (AHPRA) and (ii) a member of ASUM. Achieving CAHPU Lung POCUS accreditation involves attending a one-day ASUM-accredited lung and diaphragm course, completing the online ASUM physics image optimization units and CAHPU quiz, and completing 40 supervised scans with a suitably qualified supervisor - which also includes the completion of two formative assessment, one summative assessment, and a signed logbook. The logbook requirements and the three directly supervised assessments as described above, need to be completed and submitted within two years of attending the initial one-day Lung POCUS training course. At least 50% of the Lung POCUS scans must be clinically indicated, and 15 of those identified as a positive scan. Once successfully accredited, the physiotherapist must continue to participate in ongoing professional development and maintain an ongoing logbook of Lung POCUS scans to demonstrate ongoing competency and practice.

### Lung POCUS Accreditation Pathways for Respiratory Therapists

A recent scoping review explored the training methods of POCUS by respiratory therapists 8. Of the seven included studies that described nine different countries, the majority used a combination of educational methods such as didactic talks, hands-on sessions, and practical assessments. In terms of assessing competency, 86% of curriculums included practical assessments (e.g. 11 supervised scans at the bedside), and 43% of curriculums had a combination of theoretical and practical assessments. There is a call for the development of a respiratory therapist POCUS curriculum 8. 

### Barriers to Implementing and Gaining Lung POCUS Accreditation for Physiotherapists and Respiratory Therapists and Novel Solutions

A recent survey^13^ of physiotherapists who had attended an ASUM-accredited lung and diaphragm course reported a lack of clinical time to devote to Lung POCUS training and limited available ultrasound supervisors as the major barriers to achieving competence in performing Lung POCUS. This is similar to reports from the UK 14, in addition to other barriers including limited team support, ultrasound machine availability, and cost. A broader international survey^11^ of both physiotherapists and respiratory therapists also identified that a lack of mentors and supervisors, as well as a lack of policy, governance, support from the wider team, availability of courses and lack of ultrasound equipment, are the key barriers to the implementation of Lung POCUS. Anecdotally, within Australia, there is also suspicion that lack of clinical time and availability of mentors and supervisors for physiotherapists performing Lung POCUS means that trainees are often unable to complete the 40 Lung POCUS scans within the two-year time frame after attending the initial Lung POCUS training course. 

Collaboration and shared learning from international experiences may assist in addressing the above barriers and facilitate the overall development of Lung POCUS in physiotherapy cardiorespiratory practice. A fast-track approach of a supervised five-day Lung POCUS training programme for physiotherapists was trialled in the UK 15. This approach was sufficient to achieve successful Lung POCUS accreditation and is therefore possible with the appropriate mentee and structured training program.^16^ To the authors’ knowledge, the fast-track programme is gaining traction with physiotherapists in the UK with further results soon to be published.

Subsequently, a fast-track approach for physiotherapists in Australia has recently been trialled. This involved a shorter three-day Lung POCUS scanning programme. It averaged 15 scans per normal working day by a senior physiotherapist with prior experience in Lung POCUS to obtain CAHPU accreditation. All scans were supervised by a physiotherapist with ASUM accreditation, skilled in using Lung POCUS. This resulted in the logbook of supervised scans being completed within the three days and formal CAHPU completion within two months of registration, due to the required administrative approvals from ASUM. Additional benefits from the fast-track approach included all the 40 Lung POCUS scans being fully supervised, thus offering more opportunities for teaching and guidance, and supervision delivered by a fellow clinician enabled physiotherapy-specific interpretation of images. For the newly Lung POCUS-accredited physiotherapist, Lung POCUS was successfully implemented as part of daily practice and enabled the provision of mentorship to other local physiotherapists. This approach should be feasible within the respiratory therapy profession, but has not been formally evaluated to our knowledge.

## Conclusions

While a fast-track approach is resource intensive, such resource allocation may be justified to support the growth of Lung POCUS in physiotherapy and respiratory therapy practice where mentor support is inconsistent, lacking, or absent.Further research into the optimal delivery of the fast-track approach and the influence of Lung POCUS accreditation by physiotherapists and respiratory therapists internationally is strongly recommended. Further research into the delivery of physiotherapy and respiratory therapy Lung POCUS education, training, and implementation in clinical areas in the ICU and acute care settings should be considered a priority. 

## Disclosure Statement 

The authors declare that they have no competing interests.
